# Increase in Occurrence of Attention Deficit Hyperactivity Disorder Differs by Age Group and Gender—Finnish Nationwide Register Study

**DOI:** 10.1002/brb3.70253

**Published:** 2025-01-19

**Authors:** Elisa Westman, Tuire Prami, Alvar Kallio, Ilona Iso‐Mustajärvi, Joel Jukka, Paavo Raittinen, Maarit J Korhonen, Anita Puustjärvi, Sami Leppämäki

**Affiliations:** ^1^ Takeda Oy Helsinki Finland; ^2^ University of Helsinki Helsinki Finland; ^3^ Oriola Espoo Finland; ^4^ Kuopio University Hospital Kuopio Finland; ^5^ Terveystalo Helsinki Finland

**Keywords:** attention deficit hyperactivity disorder, incidence, medication, prevalence, register study

## Abstract

**Introduction:**

This study describes epidemiology of attention deficit hyperactivity disorder (ADHD) and use of ADHD medication across all age groups in Finland.

**Methods:**

This retrospective study is based on nationwide registers in Finland. The study population included individuals with ADHD diagnosis and/or an ADHD medication record at least once during 2015–2020.

**Results:**

The yearly prevalence of ADHD was higher in males than in females and was highest in the age groups of 6‐ to 12‐ and 13‐ to 17‐year‐old males. In 2020, the yearly prevalence was 4.2% in ≤12‐year‐old, 6.7% in 13‐ to 17‐year‐old, 0.7% in ≥18‐year‐old males, and 1.1%, 2.6%, and 0.6%, respectively, in females. The gender‐related differences were greatest among 6‐ to 12‐ and 13‐ to 17‐year‐olds, after which the differences evened out. During the study period from 2015 to 2020, the yearly prevalence more than doubled in each of the five Finnish administrative university hospital areas. The prevalence was higher in males, but the relative growth was higher in females compared to males. The incidence per 100,000 inhabitants was the highest in ≤12‐year‐old males and increased in all age groups and in both genders. The use of medication was more common in males than in females, and the overall proportion of prevalent ADHD patients on medication remained around 80%. Decrease in medication use was observed in connection with the transition from adolescence to adulthood, in both genders.

**Conclusion:**

Both prevalence and incidence of ADHD more than doubled in Finland during the study period 2015–2020. This study presents the most comprehensive analysis of national register data at personal‐level linkage in Finland, since it included all age groups, and both diagnosed ADHD patients and individuals receiving medication, not limited to reimbursed medication.

## Graphical abstract

- In Finland with total of 5.5 million inhabitants, 91,095 individuals were identified to have ADHD based on diagnosis and/or medication.
- Both prevalence and incidence of ADHD more than doubled in Finland during the study period from 2015 to 2020.

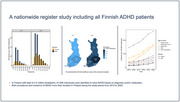

## 1 Introduction

Attention deficit hyperactivity disorder (ADHD) is a common childhood‐onset neurodevelopmental disorder, which frequently persists into adulthood (Barkley et al., 2002; Faraone et al., 2006; Kooij et al., 2019). ADHD is characterized by persistent inattention, hyperactivity, and impulsivity symptoms, which impair daily function (Adler et al., 2013; Faraone et al., 2021; Kooij et al., 2019; van Stralen, 2016). ADHD symptoms often seem to reduce in adulthood, but the burden they cause can be significant.

The global prevalence of ADHD has been estimated to be 5.9–7.2% in children (Fayyad et al., 2007; Polanczyk et al., 2015; Willcutt, 2012) and 2.2–3.4% in adults (Fayyad et al., 2007; Fayyad et al., 2017). The estimates from country‐specific studies vary (Fayyad et al., 2007; Kooij et al., 2019) and differences within a country have also been demonstrated (Widding‐Havneraas et al., 2023). Many of the studies have been based on clinical interviews or questionnaires rather than observations in administrative data, and the variability in results (range 1.2–7.3%) may be explained by methodological differences (Fayyad et al., 2007; Polanczyk et al., 2014; Willcutt, 2012).

The prevalence rates in boys are higher than in girls but the gender difference appears to almost equalize in adulthood (Nøvik et al., 2006; Nussbaum, 2012). It has been recognized that women tend to receive the diagnosis at older age than men (Grevet et al., 2006) and the use of ADHD medication is less common in girls and women compared to boys and men (Castle et al., 2007).

In Finland, the prevalence of ADHD has been estimated to be higher in children and adolescents compared to global prevalence estimates (Almqvist et al., 1999; Smalley et al., 2007). In a birth cohort study, children (*n* = 9432) born in the Northern Finland in 1986 were evaluated for ADHD behavior at the age of 16–18 by a survey, according to which the prevalence of ADHD in adolescents was estimated to be 8.5% (Smalley et al., 2007). In another birth cohort study, a sample of children (*n* = 5813) born in Finland in 1981 were assessed for ADHD symptoms at the age of 8–9 years by different screening instruments and a semistructured parent interview (Almqvist et al., 1999). Based on an older version of diagnostic criteria, the DSM‐III‐R criteria, the prevalence of ADHD was estimated to be 11.3% in boys and 2.9% in girls. Neither of these Finnish prevalence studies was, however, based on confirmed ADHD diagnoses.

The incidence of ADHD diagnoses has been shown to increase in Finland during the last decade. In register‐based analyses, the cumulative incidence of psychiatric or neurodevelopmental disorders measured from the 12th to the 18th birthday had increased over 5 percentage points in girls and 2 percentage points in boys between birth cohorts from the years 1987 and 1997 (Gyllenberg et al., 2018). The cumulative incidence was 14.9% among girls and 8.8% among boys for those born in 1997. Recent publications further support the evidence, indicating that the prevalence of ADHD among children and adolescents in Finland is higher compared to global prevalence estimates (Auro et al., 2024; Vuori et al., 2024). Increasing rates of ADHD diagnoses are suggested to reflect better diagnostic measures, increasing awareness of ADHD, increased requirements in lifestyle, and perhaps changing attitudes toward treatments (Rydell et al., 2018).

Increase in the use of ADHD medications has been observed in several countries. In the Nordic countries, the use of ADHD medication has been the highest in Iceland and lowest in Finland among children (3‐ to 18‐year‐old) (Raman et al., 2018). In the past years, several Finnish register studies have shown that the use of reimbursed ADHD medications has significantly increased. The increase has been more rapid in girls, but still the medication use is considerably lower in girls compared to boys. A recent Finnish register study showed that the annual prevalence of reimbursed ADHD medication in 2018 was 4.42% for 6‐ to 12‐year‐old and 4.21% for 13‐ to 17‐year‐old males and 0.99 % and 1.28 % in the respective age groups in girls (Vuori et al., 2020). In a more recent update from the same research group, the prevalence for reimbursed medication use had increased, and was 5.1% for boys and 2.3% girls (6–12 years old) in 2019 and 8.0% and 2.3%, respectively, in 2022 (Aalto‐Setälä and Vuori, 2023).

The aim of the present study was to describe the register‐based epidemiology of ADHD across all age groups in Finland during 2015–2020. ADHD prevalence and incidence, and use of ADHD medication were investigated and compared between age and gender groups in the five administrative Finnish healthcare areas of the university hospitals. Compared to the previously conducted studies, the present study aimed to capture all diagnoses by using both clinically confirmed diagnosis and the use of medication as a prognostic factor for a diagnosis.

## 2 Materials and Methods

This was a nationwide, retrospective register study utilizing data from national administrative databases in Finland. Nationwide Care Registers for Health Care (primary and secondary care) maintained by the Finnish Institute for Health and Welfare, as well as nationwide prescription registers including prescriptions, dispensations, and reimbursed purchases, and reimbursement register including entitlements to higher than regular medication reimbursement, of the Social Insurance Institution of Finland, were used to identify individuals with ADHD.

In this study, patients with ADHD were identified using the following criteria: ADHD‐related visit in primary or secondary health care associated with International Classification of Diseases, 10th Edition (ICD‐10) code F90* (recorded as the primary diagnosis or any of the secondary diagnoses); and/or, electronic prescription and/or (reimbursed or non‐reimbursed) purchase of ADHD medication identified by Anatomic Therapeutic Chemical (ATC) codes N06BA02, N06BA04, N06BA09, N06BA12, or C02AC02 (referring to dexamfetamine, methylphenidate, atomoxetine, lisdexamfetamine, and guanfacine, respectively); and/or, granted reimbursement code 331 for ADHD medication (taken into account during the year when the entitlement for reimbursement was granted).

In addition to diagnoses and medication information, gender, date of birth, and place of residence were captured from the above‐mentioned registers and from the Digital and Population Data Services Agency. Dates of death were obtained from the registers of Statistics Finland. Study permit was issued by the Finnish Social and Health Data Permit Authority Findata (Dnro THL/3461/14.02.00/2022). Data were stored and analyzed in a validated secure data environment provided by Statistics Finland. Statistics Finland also compiled the data sets and ensured the privacy of citizens with Findata by pseudonymizing the data. Results with frequencies < 5 individuals were not reported.

The study population consisted of Finnish ADHD patients of any age in 2015–2020. For these patients, personal‐level data were collected and linked from different sources from January 01, 2010, to December 31, 2021. The first 5 years of the data, 2010–2014, were used as a history period

Prevalent ADHD patients fulfilled any of the above‐mentioned ADHD identification criteria during 2015–2020. Incident ADHD patients were a subset of prevalent ADHD patients: An incident ADHD patient fulfilled the ADHD identification criteria for the first time during 2015–2020, and none of the criteria during the history period 2010–2014. Index date for each patient was defined as the first date when any of the identification criteria was met, and a patient was considered incident for the year when the first inclusion criterion was fulfilled. Prevalent population for a specific year consisted of patients who met any of the inclusion criteria during that calendar year. Point‐prevalence in the end of year 2020 was observed by counting patients with ADHD fulfilling any inclusion criteria any time during 2015–2020, and alive and living in Finland at the end of 2020.

Yearly numbers of prevalent and incident patients were compared against the mid‐year population count in each age and gender group in Finland to obtain prevalence per 1000 and incidence per 100,000 inhabitants. Population statistics published by Statistics Finland (including age, gender, municipality, and calendar year for aggregations) were used to calculate denominators. In regional analyses, the place of residence was ascertained for each patient for each calendar year. Whenever age was considered in prevalence analyses, the age at the end of the year was used; in incidence analyses, the age at index date was used. Chosen age groups represent different categories of specialist psychiatric care in Finland: children, 0–12 years; adolescents, 13–17 years; and adults ≥18 years. In certain analyses, an additional division into 0‐ to 5‐year‐old and 6‐ to 12‐year‐old children was made since ADHD medication is indicated for patients of 6 years and older in Finland. In these analyses also, adults of different age were divided into subcategories.

Finally, use of ADHD medications is described for both incident and prevalent ADHD patients. For incident patients, the first ADHD medication purchased at or up to 1 year after the index date was observed. Among prevalent patients for each study year, yearly prevalence of ADHD medication use was determined.

Analyses were performed using R version 4.2.2.

## 3 Results

During the study period (from 2015 to 2020), 91,095 individuals fulfilled at least one of the identification criteria for ADHD in Finland with total of 5.5 million inhabitants in 2020. The point prevalence at the end of 2020 (i.e., number of individuals with ADHD, alive and living in Finland on December 31, 2020) was 90,182. In total, 551 patients with ADHD passed away during the years 2015–2021.

**FIGURE 1:**
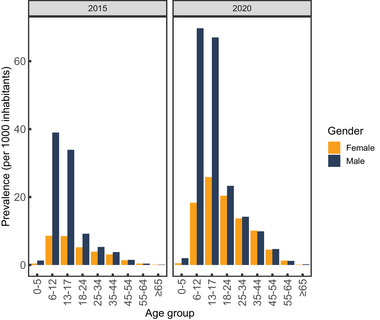
Yearly prevalence of ADHD in each age‐gender group (a) in 2015 and (b) in 2020.

The yearly prevalence of ADHD was higher in males than in females (Figure 1). By age‐gender group, the yearly prevalence for ADHD was 4.2% in 0‐ to 12‐year‐old, 6.7% in 13‐ to 17‐year‐old, 0.7% in ≥18‐year‐old males, and 1.1%, 2.6%, and 0.6%, respectively, in females in 2020. The gender‐related differences were greatest in the age groups of 6–12 years old and 13–17 years old but in adults, the differences appeared to equalize.

The yearly prevalence increased between 2015 and 2020 and the increase was relatively higher in girls and women compared to boys and men in all age groups except 0–5 years old and 65 years old and older (Figure 1). The absolute increase was the most prominent in 13‐ to 17‐year‐old adolescents in both genders. In 2020, the prevalence in females was higher in 13–17 and 18–24 years old than in 6–12 years old whereas, in males, the prevalence remained the highest in 6‐ to 12‐year‐old boys over the study period.

**FIGURE 2:**
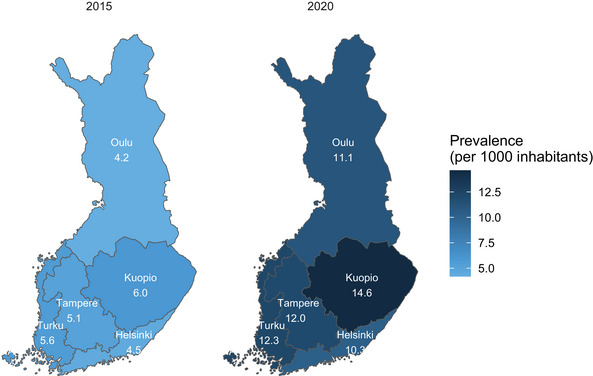
Yearly prevalence of ADHD by administrative university hospitals areas in Finland (a) in 2015 and (b) in 2020.

The yearly prevalence per 1000 inhabitants more than doubled in each of the five administrative university hospital areas in Finland during the study period (Figure 2), but the differences between areas remained approximately the same over the study period. Absolute numbers of prevalent ADHD patients each year in different age categories divided by gender is presented in Table S1. The prevalence and its increase were more prominent the northern the patient lived, especially in 13‐ to 17‐year‐old adolescents (Figure S1). Absolute numbers of prevalent ADHD patients with the respective prevalences per 1000 inhabitants in different administrative university hospital areas in 2015 and 2020 are presented in Tables S2a and b, respectively.

In total 66,146 incident ADHD patients were identified in Finland during 2015–2020 (excluding 131 individuals with no official place of residence in Finland at the time of or before the first ADHD‐related data entry). Median age at index date was 14 years: 11 years for males and 20 years for females.

**FIGURE 3:**
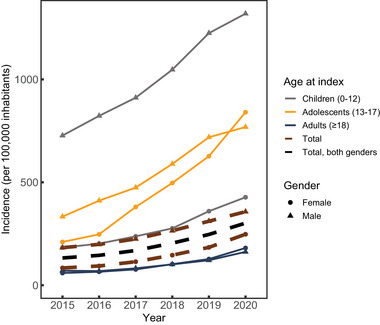
Incidence of ADHD in each age‐gender group in 2015–2020.

**FIGURE 4:**
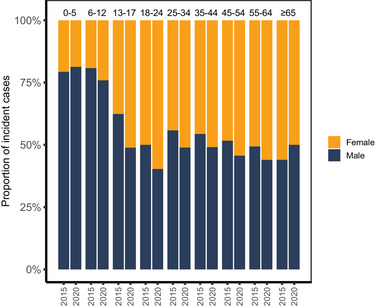
Distribution (%) of incident ADHD patients by gender in each age group in 2015 and in 2020.

Distribution of incident ADHD patients in each study year divided by age and gender is presented in Table 1. Incidence per 100,000 inhabitants is presented in Figure 3, and the respective values with absolute numbers of patients in Table S3. The incidence per 100,000 inhabitants was the highest in 0‐ to 12‐year‐old males. In 2020, it was 3.1 times higher than the respective incidence in females. During the study period, the incidence per 100,000 inhabitants increased in all age groups and in both genders. The increase was the most prominent in 13‐ to 17‐year‐old females (4.0‐fold from 2015 to 2020). In this age group, the incidence in females overtook the incidence in males in 2020. The same phenomenon but with less extent occurred in adult females compared to adult males in 2018. Similar results can be seen in Figure 4 where distributions of incident female and male ADHD patients are presented in 2015 and in 2020. In 2020, females comprised 50% or more of incident ADHD patients in all age groups of 13 years and older.

**TABLE 1:** Distribution [*n* (%)] of incident ADHD patients by gender and age in 2015–2020.

Year	2015	2016	2017	2018	2019	2020
*n*	*7259*	*7985*	*9298*	*11,285*	*13,624*	*16,695*
**Gender**						
Female	2338 (32.2)	2607 (32.6)	3193 (34.3)	4083 (36.2)	5143 (37.7)	6933 (41.5)
Male	4921 (67.8)	5378 (67.4)	6105 (65.7)	7202 (63.8)	8481 (62.3)	9762 (58.5)
**Age**						
0–5 years	401 (5.5)	379 (4.7)	454 (4.9)	460 (4.1)	469 (3.4)	556 (3.3)
6–12 years	3196 (44.0)	3666 (45.9)	4052 (43.6)	4679 (41.5)	5589 (41.0)	6009 (36.0)
13–17 years	803 (11.1)	972 (12.2)	1263 (13.6)	1609 (14.3)	2010 (14.8)	2429 (14.5)
18–24 years	808 (11.1)	879 (11.0)	1024 (11.0)	1297 (11.5)	1534 (11.3)	2057 (12.3)
25–34 years	1016 (14.0)	1088 (13.6)	1274 (13.7)	1659 (14.7)	2045 (15.0)	2924 (17.5)
35–44 years	662 (9.1)	635 (8.0)	801 (8.6)	1047 (9.3)	1292 (9.5)	1817 (10.9)
45–54 years	273 (3.8)	277 (3.5)	312 (3.4)	397 (3.5)	539 (4.0)	666 (4.0)
55–64 years	75 (1.0)	74 (0.9)	91 (1.0)	117 (1.0)	122 (0.9)	191 (1.1)
≥65 years	25 (0.3)	15 (0.2)	27 (0.3)	20 (0.2)	24 (0.2)	46 (0.3)
Children (0–12 years)	3597 (49.6)	4045 (50.7)	4506 (48.5)	5139 (45.5)	6058 (44.5)	6565 (39.3)
Adolescents (13–17 years)	803 (11.1)	972 (12.2)	1263 (13.6)	1609 (14.3)	2010 (14.8)	2429 (14.5)
Adults (≥18 years)	2859 (39.4)	2968 (37.2)	3529 (38.0)	4537 (40.2)	5556 (40.8)	7701 (46.1)

The first purchased ADHD medications at or within 1 year after the index date (for the incident patients from year 2020 the follow‐up time continued to year 2021) are listed in Table 2a. The most common first ADHD medication in the incident ADHD population was methylphenidate; the other ADHD medications were used less often as the initial treatment. In total, 76% of the incident patients received methylphenidate and 24% did not receive any ADHD medication within 1 year from the index date. When observing the prevalent ADHD population during different study years (i.e., including patients with diagnosis or medication records in a particular year), methylphenidate was used by 74% in 2015 and by 70% in 2020 (Table 2b). At the same time, the prevalence of lisdexamfetamine use increased from 3% to 11%. In this analysis, the proportion of ADHD patients with any medication remained stable, about 80%, over the study period. These yearly results of medication use by gender and region are presented in Tables S4a and b, respectively.

**TABLE 2a:** The first ADHD medication [*n* (%)] at or within 1 year after the index date in incident ADHD patients by age.

Age	Children (0–12 years)	Adolescents (13–17 years)	Adults (≥ 18 years)	Total
**Medication**				
Methylphenidate	22,615 (75.6)	6961 (76.6)	18,622 (68.6)	48,198 (72.9)
Atomoxetine	92 (0.3)	289 (3.2)	1210 (4.5)	1591 (2.4)
Lisdexamfetamine	24 (0.1)	33 (0.4)	436 (1.6)	493 (0.7)
Guanfacine	23 (0.1)	25 (0.3)	16 (0.1)	64 (0.1)
Dexamfetamine	< 5	< 5	55 (0.2)	
No medication	7159 (23.9)	1778 (19.6)	6817 (25.1)	15,754 (23.8)

**TABLE 2b:** Prevalence of ADHD medication use [*n* (% of prevalent ADHD population)] by calendar year and age group.

		Year					
Medication	Age group (years)	2015	2016	2017	2018	2019	2020)
*n*		*27,164*	*31,582*	*37,098*	*44,297*	*53,045*	*64,393*
Methylphenidate	Children (0–12)	7828 (74.8)	8790 (74.0)	9932 (74.5)	11,410 (74.8)	13,080 (74.9)	14,796 (75.1)
Methylphenidate	Adolescents (13–17)	4799 (76.0)	5394 (74.3)	6341 (74.3)	7428 (72.7)	8729 (72.6)	10,116 (71.4)
Methylphenidate	Adults (≥ 18)	7374 (71.1)	8618 (69.3)	10,540 (69.2)	13,009 (69.1)	15,868 (67.4)	19,961 (65.4)
Methylphenidate	Total	20,001 (73.6)	22,802 (72.2)	26,813 (72.3)	31,847 (71.9)	37,677 (71.0)	44,873 (69.7)
Lisdexamfetamine	Children (0–12)	274 (2.6)	486 (4.1)	734 (5.5)	1045 (6.9)	1291 (7.4)	1593 (8.1)
Lisdexamfetamine	Adolescents (13–17)	101 (1.6)	261 (3.6)	462 (5.4)	718 (7.0)	961 (8.0)	1316 (9.3)
Lisdexamfetamine	Adults (≥ 18)	303 (2.9)	610 (4.9)	980 (6.4)	1661 (8.8)	2797 (11.9)	4456 (14.6)
Lisdexamfetamine	Total	678 (2.5)	1357 (4.3)	2176 (5.9)	3424 (7.7)	5049 (9.5)	7365 (11.4)
Atomoxetine	Children (0–12)	722 (6.9)	706 (5.9)	771 (5.8)	853 (5.6)	871 (5.0)	1016 (5.2)
Atomoxetine	Adolescents (13–17)	568 (9.0)	604 (8.3)	689 (8.1)	802 (7.8)	903 (7.5)	1025 (7.2)
Atomoxetine	Adults (≥ 18)	715 (6.9)	768 (6.2)	874 (5.7)	1016 (5.4)	1213 (5.1)	1754 (5.7)
Atomoxetine	Total	2005 (7.4)	2078 (6.6)	2334 (6.3)	2671 (6.0)	2987 (5.6)	3795 (5.9)
Dexamfetamine	Children (0–12)	0 (0.0)	14 (0.1)	37 (0.3)	43 (0.3)	52 (0.3)	53 (0.3)
Dexamfetamine	Adolescents (13–17)	0 (0.0)	9 (0.1)	10 (0.1)	25 (0.2)	29 (0.2)	44 (0.3)
Dexamfetamine	Adults (≥ 18)	< 5 (0.0)	311 (2.5)	485 (3.2)	592 (3.1)	754 (3.2)	973 (3.2)
Dexamfetamine	Total	< 5 (0.0)	334 (1.1)	532 (1.4)	660 (1.5)	835 (1.6)	1070 (1.7)
Guanfacine	Children (0–12)	0 (0.0)	49 (0.4)	137 (1.0)	173 (1.1)	241 (1.4)	333 (1.7)
Guanfacine	Adolescents (13–17)	0 (0.0)	14^†^ (0.2)	58 (0.7)	102 (1.0)	165 (1.4)	228 (1.6)
Guanfacine	Adults (≥ 18)	0 (0.0)	< 5^†^ (0.0)	23 (0.2)	23 (0.1)	53 (0.2)	75 (0.2)
Guanfacine	Total	0 (0.0)	63 (0.2)	218 (0.6)	298 (0.7)	459 (0.9)	636 (1.0)
Any ADHD medication	Children (0–12)	8221 (78.5)	9355 (78.7)	10,653 (79.9)	12,314 (80.8)	14,210 (81.4)	16,114 (81.8)
Any ADHD medication	Adolescents (13–17)	5229 (82.8)	5954 (82.0)	7094 (83.1)	8520 (83.4)	10,047 (83.5)	11,810 (83.3)
Any ADHD medication	Adults (≥ 18)	7993 (77.0)	9567 (76.9)	11,887 (78.1)	14,754 (78.3)	18,529 (78.7)	23,958 (78.5)
Any ADHD medication	Total	21,443 (78.9)	24,876 (78.8)	29,634 (79.9)	35,588 (80.3)	42,786 (80.7)	51,882 (80.6)

^†^ Value < 5 in 2016 has been included in the age group 13–17 so that it cannot be calculated using other reported information.

**FIGURE 5:**
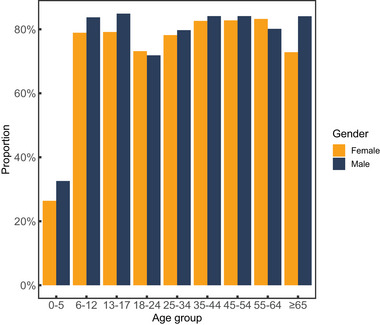
Proportion (%) of prevalent male and female ADHD patients receiving ADHD medication in 2020.

The use of ADHD medication was most common in males 13–17 years old (84.9%) compared to other male or any female age groups in 2020 (Figure 5). In females, the use of ADHD medication was the most common in middle age (more than 82% in the age groups covering 35‐ to 64‐year‐old individuals). In both gender groups, a decline in medication use was seen in connection with the transition from adolescence to adulthood.

## 4 Discussion

This 6‐year register study showed that both prevalence and incidence of ADHD more than doubled in Finland during the study period, from 2015 to 2020. As far as we know, our study describes the nationwide prevalence and incidence of ADHD across all age groups to this extent in Finland for the first time. The prevalence was higher in males than in females, but the increase of prevalence was notably higher in females (Figure 1). In adolescents and adults, the increase in prevalence was more than threefold in females and approximately twofold in males. The incidence of ADHD increased in all age groups and was the highest in male children (Figure 3). Similarly to prevalence, the relative increase in incidence was higher in females than in males, and it was the most prominent in 13‐ to 17‐year‐old females. These findings were supported in a recent publication, which used the same nationwide registers as in our study (Auro et al., 2024).

Earlier, it was assumed that the prevalence of ADHD would be somewhat similar between continents and countries. Higher incidence rates compared to previous global estimates and increase in the use of ADHD medication have raised concerns about overdiagnosis or overprescribing (Aalto‐Setälä and Vuori, 2023; Thomas et al., 2015). Given that the previous prevalence studies mainly focused on descriptive prevalence in children and adolescents, there has been lack of evidence demonstrating prevalence of ADHD based on combination of clinical diagnosis and use of medication among all age groups. The newest estimates suggest the global prevalence to be approximately 8% for children and adolescents, and twice as high in boys (10%) compared to girls (5%) (Ayano et al., 2023; Salari et al., 2023). Many of the prevalence estimates are based on meta‐analyses from clinical studies or symptom questionnaires without actual diagnoses. Register data allows us to evaluate clinically confirmed ADHD cases, and the present study further confirms that the prevalence of ADHD in Finland is moderate compared to global estimates.

In addition to between‐country differences, even 10‐fold (2–20%) within‐country differences in male children and adolescent have been reported in Finland (Vuori et al., 2024). Similar regional variability was seen in the present study, although the division to geographical areas was done on a coarser level. We believe, these remarkable areal differences most likely demonstrate not only true clinical but also technical differences related to data records.

Timewise, we saw a large increase in incidence and prevalence of ADHD from 2015 to 2020, and with a more up‐to‐date dataset would have most likely seen a similarly prominent increase after 2020, too. Our study can be used as a reference representing the prepandemic years when later evaluating the effect of the pandemia on ADHD treatment. For example, availability of psychosocial support was most likely poorer during the pandemic leading to delays in ADHD diagnoses and distress in patients waiting for evaluation. It is notable that the yearly prevalence in 2020 was about 64,000 (Table 2b) and it differs remarkably from the cumulative point prevalence at the end of 2020: about 90,000 Finnish ADHD patients. This means that about one third of all individuals identified with ADHD during 2015–2020 did not have any ADHD‐related contacts with the health care providers (no visit or medication records) in 2020.

The nationwide care register for primary care visits, AvoHilmo, was launched in 2011 (Mölläri and Saukkonen, 2024). Before that, contacts at primary care were not recorded to nationwide administrative registers. In addition, outpatient contacts at private health care, which in great part organizes occupational health care in Finland, have not been comprehensively covered by nationwide registers until 2020 (Suvisaari, 2021). In general, the launch of AvoHilmo and inclusion of private health care data have improved the coverage of the nationwide administrative health care registers in Finland. However, during launching period, which overlaps with our study history period and when AvoHilmo data were not comprehensive, some ADHD diagnoses from primary care may be missing in our data. The same limitation applies to diagnoses made in private healthcare before 2020. These factors may have led to overestimation of incident and underestimation of prevalent ADHD cases in our study. To overcome the limitation, medication use without diagnosis was considered as diagnosis in this study since prescription and dispensation data are more comprehensive.

In Finland, ADHD medications are used off‐label for treating other conditions such as narcolepsy, traumatic brain injury, and binge eating disorder. Narcolepsy was the most common but still very rare condition (0.5% of all identified ADHD patients) in our data. Patients with these possible off‐label indications were not excluded from the study population as their inclusion has only a minor effect on the results. It is also notable that patients with these indications may have had ADHD too.

In the present Finnish data, the most common first ADHD medication in the incident ADHD population was methylphenidate. This is not surprising since according to the national treatment guideline, methylphenidate is the preferred medication choice for treating ADHD (ADHD (attention deficit hyperactivity disorder): Current Care Guidelines 2019). This is further enhanced by the national reimbursement criteria, which require a separate reimbursement application to all other ADHD medication than methylphenidate (The Social Insurance Institution of Finland 2024). The slight increase of lisdexamfetamine use during the study period can be explained by the fact that lisdexamfetamine became available just before the study period, midyear 2014.

Proportion of prevalent ADHD patients receiving medication each year remained around 80% throughout the study period although the prevalence of ADHD more than doubled during that time (Table 2b). The proportion of patients using medication in Finland (Raman et al., 2018) has been shown to be slightly lower compared to what has been published, for example, from the Swedish national registers: between years 2018 and 2021, 84.5% of patients received medication or at least prescription for medication in Sweden (Giacobini et al., 2018), and no incremental increase in ADHD medication use exceeding the increase in new ADHD diagnoses was seen between years 2015 and 2022 (Auro et al., 2024). In the Swedish data, the prescriptions were more common in adults compared to children and in males compared to females. In our study, the use of medication was higher among 13‐ to 17‐year‐old boys compared to men in any other age (Figure 5). The use of medication was more common in males compared to females in almost every age group, with an exception that in the age groups of 18–24 years old and 55–64 years old the proportion of women on medication was slightly higher than in the males of the same age. A recent Finnish study showed that the increase in medication use was greater in girls compared to boys (aged 6–18 years old) during 2008–2019 (Kolari et al., 2024).

Interestingly we found that there was a drop‐down in the use of medication between the age groups of 13‐ to 17‐year‐old adolescents and 18‐ to 24‐year‐old young adults. This was evident in both genders but more remarkable in males (Figure 5). This could be related to the organization of healthcare services in Finland, where over 18‐year‐old patients should be transitioned from adolescent psychiatry departments to adult psychiatry departments. This transition between service providers may cause temporary interruption of treatment and should be considered when organizing local psychiatric treatment paths.

Another explanation for the decrease in medication use could be that all Finnish male citizens from 18 years of age onward are obligated to take part in the military defense (in either armed or unarmed service) (The Finnish Defence Forces 2024). Patients with ADHD will receive exemption from military service if their ADHD causes significant functional impairment or requires continuous medication. This restriction in the use of medication may lead to discontinuation of treatment if young men wish to perform their military service. In 2022, 74% of the male population born in 2004 (about 23,000 males) were assigned for military service. In addition, about 1500 women applied for voluntary military service in Finland in 2022 (The Finnish Defence Forces 2022). The same year, only 1.6% of the men chose unarmed service, and 8.9% of the men had an exemption from military service due to health issues.

## 5 Conclusion

To our knowledge, this study presents the most comprehensive analysis of national register data in Finland, since it included both patients with ADHD diagnosis and individuals receiving medication, not limited to reimbursed medication and it included all age groups.

This 6‐year register study showed that both prevalence and incidence of ADHD more than doubled in Finland from 2015 to 2020. Incidence and prevalence were higher in males than in females and was the highest in young males. The relative growth was higher in females compared to males.

The use of medication was higher among males than females. The overall proportion of prevalent ADHD patients receiving medication remained around 80% throughout the study period although the prevalence itself more than doubled during that time.

An interesting decrease in medication use was observed between adolescents and adults in both genders. Further research is needed to understand the possible struggles related to transition from adolescent psychiatry departments to adult psychiatry departments.

## Author Contributions

Conceptualization: EW, JJ, AP, SL. Data curation: AK, PR. Formal analysis: AK, PR. Funding acquisition: EW, JJ. Investigation: EW, TP, II, JJ, MJK, AP, SL. Methodology: TP, PR, MJK. Project administration: EW, II. Resources: EW, II. Software: AK, PR. Supervision: MJK, AP, SL. Validation: PR, MJK. Visualization: AK. Writing–original draft: EW, TP, AK, PR. Writing–review & editing: II, JJ, MJK, AP, SL. All authors have contributed to the manuscript substantially and fully meet the criteria 1–5 of the International Committee of Medical Journal Editors (ICMJE) for authorship. They have agreed to the final submitted version.

## Patient Consent Statement

According to local legislation, informed consents were not required due to the secondary nature of the register data.

## Ethics Statement

Study permit was granted by Finnish Social and Health Data Permit Authority Findata with diary number THL/3461/14.02.00/2022. Study protocol was subjected to ethical consideration during the Findata permit process. No separate ethical committee process was required according to local legislation.

## Conflicts of Interest

EW and JJ are employees of Takeda Oy but have no Takeda stock or stock options. TP, AK, II, PR, and MJK declare no conflicts of interest. AP has received consultation fees from Takeda and Biocodex, and SL from Takeda, Lundbeck, and Janssen.

## Reproduction of Copyright Material

No excerpts from copyrighted works owned by third parties are included.

### Peer Review

The peer review history for this article is available at https://publons.com/publon/10.1002/brb3.70253.

## SIGNIFICANT OUTCOMES

• Prevalence and incidence of ADHD have increased among all age and gender groups, but the proportion of prevalent ADHD patients on ADHD medication has remained similar during the recent years. Compared to global estimates, ADHD prevalence is moderate in Finland.

• Increase of prevalence and incidence of ADHD was considerably higher in females compared to males.

• Proportion of prevalent ADHD patients receiving medication each year remained around 80% throughout the study period 2015–2020 although the prevalence of ADHD more than doubled during that time.

## LIMITATIONS

• Unlike prevalence estimates based on symptom questionnaires, this study excludes patients without clinically confirmed diagnosis. Therefore, these estimates are not entirely comparable. However, the study population includes patients identified from both patient and prescription registers making the inclusion more comprehensive.

• This study includes data obtained from the Finnish national registers, which have limitations related to the coverage of private healthcare data. Thus, the patients diagnosed at primary care or visiting private sector (mostly adults) may remain outside our analyses unless they have medication purchases.

• As the coverage of private and primary health care data in the Finnish national registers improved during the study period, some prevalent ADHD patients may have been misclassified as incident cases.

## Supporting information




**Figure S1**: Yearly prevalence of ADHD by administrative university hospital areas in Finland in 2015 and in 2020: (a, b) in 6‐ to 12‐year‐old children and (c, d) in 13‐ to 17‐year‐old adolescents.

**Table S1**: Absolute number of prevalent ADHD patients in each age‐gender group by calendar year.
**Table S2a**: Prevalence of ADHD [absolute *n* (per 1000 inhabitants)] by age‐gender groups and administrative university hospital areas in 2015.
**Table S2b**: Prevalence of ADHD [absolute *n* (per 1000 inhabitants)] by age‐gender groups and administrative university hospital areas in 2020.
**Table S3**: Incidence of ADHD [absolute *n* (per 100,000 inhabitants)] by age‐gender groups and calendar years.
**Table S4a**: Prevalence of ADHD medication use [*n* (% of yearly prevalent ADHD population)] by genders and years.
**Table S4b**: Prevalence of ADHD medication use [*n* (% of yearly prevalent ADHD population)] by calendar year and administrative university hospital areas.

## Data Availability

The data that support the findings of this study are available from Finnish Social and Health Data Permit Authority Findata. Restrictions apply to the availability of these data, which were used under license for this study. The data can be received with permission of Findata: https://findata.fi/en/permits/.
