# Supplementary figures and images for: Increase in Occurrence of Attention Deficit Hyperactivity Disorder Differs by Age Group and Gender—Finnish Nationwide Register Study
Source: Brain Behav. 2025 Jan 19;15(1):e70253. doi: 10.1002/brb3.70253 (PMC11743994; doi:10.1002/brb3.70253)

2015

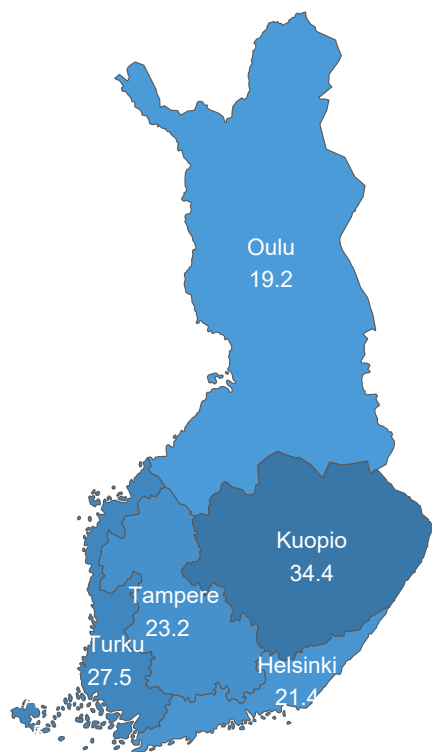

2020

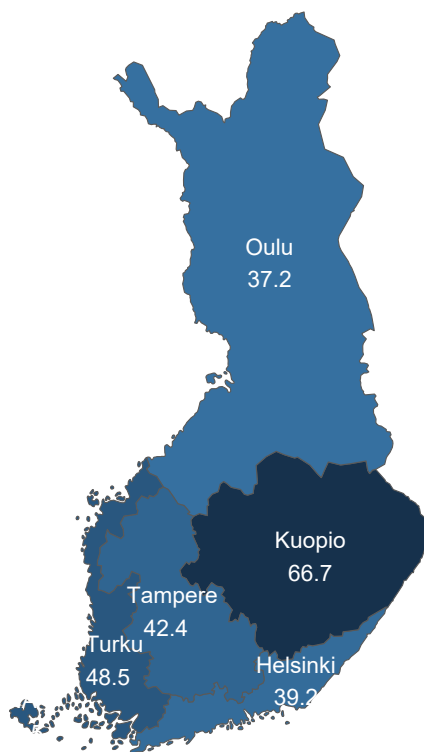

Prevalence  
(per 1000 inhabitants)

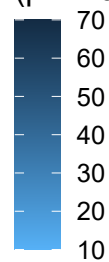

Supplement: Supplementary file 1 — Figure S1: Yearly prevalence of ADHD by administrative university hospital areas in Finland in 2015 and in 2020: (a, b) in 6‐ to 12‐year‐old children and (c, d) in 13‐ to 17‐year‐old adolescents. [file BRB3-15-e70253-s002.zip › Supp_Figure_1ab.pdf]

2015

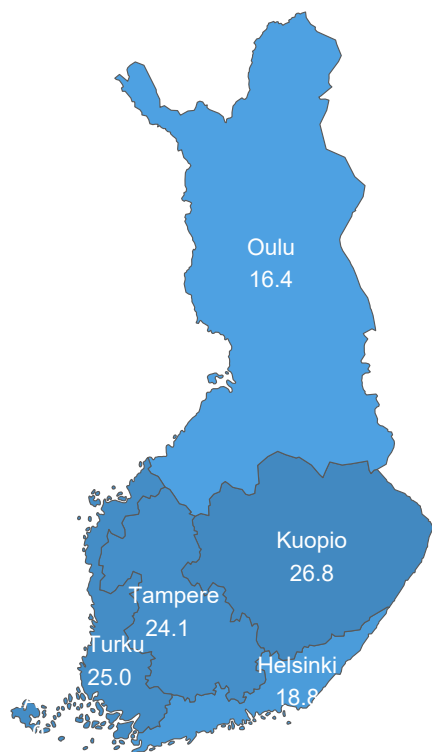

2020

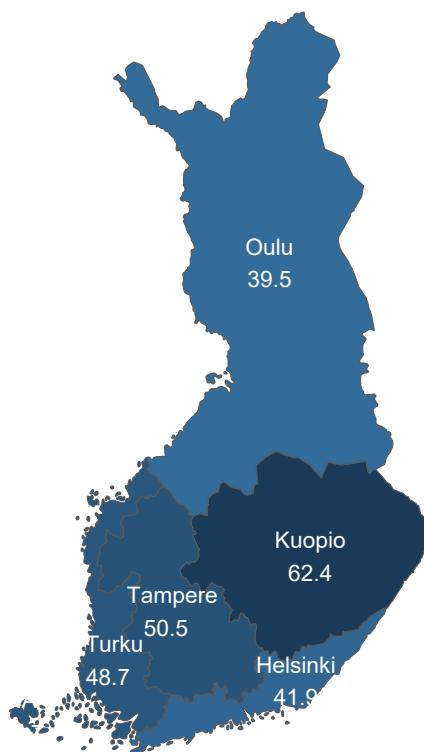

Prevalence  
(per 1000 inhabitants)

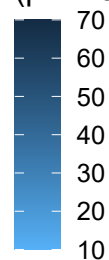

Supplement: Supplementary file 1 — Figure S1: Yearly prevalence of ADHD by administrative university hospital areas in Finland in 2015 and in 2020: (a, b) in 6‐ to 12‐year‐old children and (c, d) in 13‐ to 17‐year‐old adolescents. [file BRB3-15-e70253-s002.zip › Supp_Figure_1cd.pdf]
