# Supplementary material for: Increase in Occurrence of Attention Deficit Hyperactivity Disorder Differs by Age Group and Gender—Finnish Nationwide Register Study
Source: Brain Behav. 2025 Jan 19;15(1):e70253. doi: 10.1002/brb3.70253 (PMC11743994; doi:10.1002/brb3.70253)
Supplement: Supplementary file 2 — Table S1: Absolute number of prevalent ADHD patients in each age‐gender group by calendar year. Table S2a: Prevalence of ADHD [absolute n (per 1000 inhabitants)] by age‐gender groups and administrative university hospital areas in 2015. Table S2b: Prevalence of ADHD [absolute n (per 1000 inhabitants)] by age‐gender groups and administrative university hospital areas in 2020. Table S3: Incidence of ADHD [absolute n (per 100,000 inhabitants)] by age‐gender groups and calendar years. Table S4a: Prevalence of ADHD medication use [n (% of yearly prevalent ADHD population)] by genders and years. Table S4b: Prevalence of ADHD medication use [n (% of yearly prevalent ADHD population)] by calendar year and administrative university hospital areas. [file BRB3-15-e70253-s001.docx]

SUPPORTING INFORMATION TABLES

**Supporting Information Table 1:** Absolute number of prevalent ADHD patients in each age-gender group by calendar year

|  |  |  |  | **Year** | |  |  |
| --- | --- | --- | --- | --- | --- | --- | --- |
| **Age, years** | **Gender** | **2015** | **2016** | **2017** | **2018** | **2019** | **2020** |
| 0–5 | Female | 69 | 62 | 71 | 82 | 67 | 72 |
| 0–5 | Male | 247 | 253 | 284 | 281 | 283 | 310 |
| 6–12 | Female | 1779 | 2053 | 2331 | 2724 | 3284 | 3883 |
| 6–12 | Male | 8374 | 9512 | 10652 | 12157 | 13829 | 15443 |
| 13–17 | Female | 1221 | 1450 | 1855 | 2429 | 2970 | 3832 |
| 13–17 | Male | 5096 | 5812 | 6684 | 7790 | 9057 | 10341 |
| 18–24 | Female | 1189 | 1457 | 1812 | 2359 | 3082 | 4251 |
| 18–24 | Male | 2196 | 2464 | 2993 | 3506 | 4197 | 5160 |
| 25–34 | Female | 1310 | 1715 | 2124 | 2667 | 3418 | 4678 |
| 25–34 | Male | 1906 | 2214 | 2607 | 3205 | 4037 | 5163 |
| 35–44 | Female | 1012 | 1267 | 1669 | 2105 | 2655 | 3464 |
| 35–44 | Male | 1301 | 1586 | 1875 | 2353 | 2852 | 3606 |
| 45–54 | Female | 492 | 578 | 719 | 892 | 1192 | 1486 |
| 45–54 | Male | 561 | 674 | 832 | 1004 | 1256 | 1580 |
| 55–64 | Female | 169 | 188 | 232 | 298 | 353 | 471 |
| 55–64 | Male | 164 | 208 | 250 | 313 | 352 | 448 |
| ≥65 | Female | 37 | 43 | 52 | 62 | 75 | 92 |
| ≥65 | Male | 41 | 46 | 56 | 70 | 86 | 113 |
| Total | Female | 7278 | 8813 | 10865 | 13618 | 17096 | 22229 |
| Total | Male | 19886 | 22769 | 26233 | 30679 | 35949 | 42164 |
| Total | Total | 27164 | 31582 | 37098 | 44297 | 53045 | 64393 |

**Supporting Information Table 2a:** Prevalence of ADHD [absolute n (per 1000 inhabitants)] by age-gender groups and administrative university hospital areas in 2015

|  |  | **Age, years^†^** | | | | | | |
| --- | --- | --- | --- | --- | --- | --- | --- | --- |
| **Region** | **Gender** | **6–12** | **13–17** | **18–24** | **25–34** | **35–44** | **45–54** | **55–64** |
| Helsinki | Female | 598 (7.5) | 400 (7.3) | 427 (4.8) | 504 (3.4) | 388 (2.8) | 212 (1.4) | 74 (0.5) |
| Helsinki | Male | 2867 (34.8) | 1690 (29.9) | 753 (8.3) | 685 (4.6) | 513 (3.6) | 250 (1.7) | 62 (0.5) |
| Tampere | Female | 279 (8.2) | 239 (10.1) | 193 (5.4) | 195 (3.6) | 185 (3.6) | 86 (1.5) | 27 (0.4) |
| Tampere | Male | 1339 (37.6) | 929 (37.4) | 411 (10.6) | 297 (5.2) | 205 (3.8) | 110 (1.8) | 36 (0.6) |
| Turku | Female | 335 (10.4) | 234 (10.1) | 228 (6.1) | 243 (4.6) | 193 (3.8) | 91 (1.6) | 33 (0.5) |
| Turku | Male | 1490 (43.8) | 959 (38.9) | 422 (10.7) | 365 (6.5) | 245 (4.6) | 94 (1.6) | 27 (0.5) |
| Oulu | Female | 196 (6.2) | 138 (6.5) | 132 (4.3) | 152 (3.6) | 83 (2.1) | 40 (0.9) | 17 (0.3) |
| Oulu | Male | 1036 (31.5) | 578 (25.7) | 253 (7.5) | 226 (4.8) | 154 (3.6) | 53 (1.1) | 20 (0.4) |
| Kuopio | Female | 371 (13.0) | 210 (10.0) | 209 (6.1) | 216 (4.9) | 163 (3.9) | 63 (1.2) | 18 (0.3) |
| Kuopio | Male | 1642 (54.9) | 940 (43.0) | 357 (9.9) | 333 (6.8) | 184 (4.2) | 54 (1.0) | 19 (0.3) |
| Total | Female | 1779 (8.6) | 1221 (8.5) | 1189 (5.2) | 1310 (3.9) | 1012 (3.1) | 492 (1.4) | 169 (0.4) |
| Total | Male | 8374 (39.0) | 5096 (33.9) | 2196 (9.2) | 1906 (5.3) | 1301 (3.8) | 561 (1.5) | 164 (0.4) |

^†^ Age categories 0–5 and ≥65 have been removed to protect privacy of people in groups with N<5.

**Supporting Information Table 2b:** Prevalence of ADHD [absolute n (per 1000 inhabitants)] by age-gender groups and administrative university hospital areas in 2020

|  |  | **Age, years^†^** | | | | | | |
| --- | --- | --- | --- | --- | --- | --- | --- | --- |
| **Region** | **Gender** | **6–12** | **13–17** | **18–24** | **25–34** | **35–44** | **45–54** | **55–64** |
| Helsinki | Female | 1253 (15.1) | 1367 (23.7) | 1368 (16.7) | 1789 (11.7) | 1281 (8.6) | 564 (4.1) | 197 (1.4) |
| Helsinki | Male | 5427 (62.1) | 3560 (59.6) | 1639 (19.4) | 1861 (11.8) | 1326 (8.4) | 648 (4.6) | 175 (1.3) |
| Tampere | Female | 619 (17.8) | 648 (26.5) | 770 (22.9) | 776 (14.7) | 634 (11.5) | 268 (5.1) | 73 (1.2) |
| Tampere | Male | 2402 (66.0) | 1872 (73.6) | 936 (25.6) | 816 (14.4) | 627 (10.8) | 276 (5.0) | 65 (1.1) |
| Turku | Female | 696 (21.0) | 608 (26.3) | 728 (21.2) | 810 (15.6) | 593 (11.0) | 256 (4.8) | 87 (1.5) |
| Turku | Male | 2596 (74.9) | 1712 (69.9) | 945 (25.8) | 912 (16.1) | 642 (11.3) | 266 (4.8) | 97 (1.7) |
| Oulu | Female | 482 (15.0) | 471 (21.4) | 571 (20.9) | 601 (14.6) | 451 (10.6) | 181 (4.6) | 46 (0.9) |
| Oulu | Male | 1966 (58.6) | 1320 (56.7) | 668 (21.5) | 688 (15.1) | 446 (9.7) | 167 (3.9) | 50 (1.0) |
| Kuopio | Female | 833 (29.2) | 738 (36.0) | 814 (26.3) | 702 (16.2) | 505 (11.6) | 217 (4.9) | 68 (1.2) |
| Kuopio | Male | 3052 (102.8) | 1877 (87.5) | 972 (30.0) | 886 (18.3) | 565 (12.1) | 223 (4.9) | 61 (1.0) |
| Total | Female | 3883 (18.3) | 3832 (25.9) | 4251 (20.4) | 4678 (13.7) | 3464 (10.1) | 1486 (4.5) | 471 (1.3) |
| Total | Male | 15443 (69.7) | 10341 (67.0) | 5160 (23.3) | 5163 (14.2) | 3606 (9.9) | 1580 (4.7) | 448 (1.2) |

^†^ Age categories 0–5 and ≥65 have been removed to protect privacy of people in groups with N<5.

**Supporting Information Table 3:** Incidence of ADHD [absolute n (per 100,000 inhabitants)] by age-gender groups and calendar years

|  |  |  | |  | | **Year** | |  | |  | |
| --- | --- | --- | --- | --- | --- | --- | --- | --- | --- | --- | --- |
| **Age at index date (years)** | **Gender** | **2015** | **2016** | **2017** | **2018** | | **2019** | | **2020** | |  |
| Children (0–12) | Female | 697 (182.7) | 769 (202.0) | 900 (237.7) | 1035 (276.3) | | 1327 (359.6) | | 1552 (427.4) | |  |
| Children (0–12) | Male | 2900 (727.2) | 3276 (822.8) | 3606 (911.1) | 4104 (1047.3) | | 4731 (1224.6) | | 5013 (1319.4) | |  |
| Adolescents (13–17) | Female | 302 (210.1) | 354 (247.3) | 546 (380.3) | 717 (496.8) | | 914 (626.8) | | 1242 (840.3) | |  |
| Adolescents (13–17) | Male | 501 (333.3) | 618 (410.9) | 717 (474.3) | 892 (589.2) | | 1096 (718.9) | | 1187 (768.5) | |  |
| Adults (≥18) | Female | 1339 (59.3) | 1484 (65.5) | 1747 (76.9) | 2331 (102.4) | | 2902 (127.2) | | 4139 (180.9) | |  |
| Adults (≥18) | Male | 1520 (70.8) | 1484 (68.8) | 1782 (82.2) | 2206 (101.3) | | 2654 (121.4) | | 3562 (162.2) | |  |
| Total | Female | 2338 (84.0) | 2607 (93.5) | 3193 (114.3) | 4083 (146.1) | | 5143 (184.0) | | 6933 (247.7) | |  |
| Total | Male | 4921 (182.5) | 5378 (198.7) | 6105 (224.8) | 7202 (264.7) | | 8481 (311.1) | | 9762 (357.4) | |  |
| Total | Total | 7259 (132.5) | 7985 (145.3) | 9298 (168.8) | 11285 (204.6) | | 13624 (246.7) | | 16695 (301.9) | |  |

**Supporting Information Table 4a:** Prevalence of ADHD medication use [n (% of yearly prevalent ADHD population)] by genders and years

|  |  | **Year** | | | | | |
| --- | --- | --- | --- | --- | --- | --- | --- |
| **Medication** | **Gender** | **2015** | **2016** | **2017** | **2018** | **2019** | **2020** |
| Methylphenidate | Female | 5307 (72.9) | 6222 (70.6) | 7720 (71.1) | 9660 (70.9) | 11849 (69.3) | 15158 (68.2) |
| Methylphenidate | Male | 14694 (73.9) | 16580 (72.8) | 19093 (72.8) | 22187 (72.3) | 25828 (71.8) | 29715 (70.5) |
| Methylphenidate | Total | 20001 (73.6) | 22802 (72.2) | 26813 (72.3) | 31847 (71.9) | 37677 (71.0) | 44873 (69.7) |
| Lisdexamfetamine | Female | 179 (2.5) | 352 (4.0) | 575 (5.3) | 994 (7.3) | 1652 (9.7) | 2638 (11.9) |
| Lisdexamfetamine | Male | 499 (2.5) | 1005 (4.4) | 1601 (6.1) | 2430 (7.9) | 3397 (9.4) | 4727 (11.2) |
| Lisdexamfetamine | Total | 678 (2.5) | 1357 (4.3) | 2176 (5.9) | 3424 (7.7) | 5049 (9.5) | 7365 (11.4) |
| Atomoxetine | Female | 480 (6.6) | 557 (6.3) | 635 (5.8) | 754 (5.5) | 887 (5.2) | 1258 (5.7) |
| Atomoxetine | Male | 1525 (7.7) | 1521 (6.7) | 1699 (6.5) | 1917 (6.2) | 2100 (5.8) | 2537 (6.0) |
| Atomoxetine | Total | 2005 (7.4) | 2078 (6.6) | 2334 (6.3) | 2671 (6.0) | 2987 (5.6) | 3795 (5.9) |
| Dexamfetamine | Female | 0 (0.0) | 108 (1.2) | 170 (1.6) | 236 (1.7) | 304 (1.8) | 404 (1.8) |
| Dexamfetamine | Male | <5 (0.0) | 226 (1.0) | 362 (1.4) | 424 (1.4) | 531 (1.5) | 666 (1.6) |
| Dexamfetamine | Total | <5 (0.0) | 334 (1.1) | 532 (1.4) | 660 (1.5) | 835 (1.6) | 1070 (1.7) |
| Guanfacine | Female | 0 (0.0) | 7 (0.1) | 26 (0.2) | 32 (0.2) | 73 (0.4) | 109 (0.5) |
| Guanfacine | Male | 0 (0.0) | 56 (0.2) | 192 (0.7) | 266 (0.9) | 386 (1.1) | 527 (1.2) |
| Guanfacine | Total | 0 (0.0) | 63 (0.2) | 218 (0.6) | 298 (0.7) | 459 (0.9) | 636 (1.0) |
| Any ADHD medication | Female | 5656 (77.7) | 6782 (77.0) | 8476 (78.0) | 10694 (78.5) | 13380 (78.3) | 17434 (78.4) |
| Any ADHD medication | Male | 15787 (79.4) | 18094 (79.5) | 21158 (80.7) | 24894 (81.1) | 29406 (81.8) | 34448 (81.7) |
| Any ADHD medication | Total | 21443 (78.9) | 24876 (78.8) | 29634 (79.9) | 35588 (80.3) | 42786 (80.7) | 51882 (80.6) |

**Supporting Information Table 4b:** Prevalence of ADHD medication use [n (% of yearly prevalent ADHD population)] by calendar year and administrative university hospital areas

|  |  | **Year** | | | | | |
| --- | --- | --- | --- | --- | --- | --- | --- |
| **Medication** | **Region** | **2015** | **2016** | **2017** | **2018** | **2019** | **2020** |
| Methylphenidate | Helsinki | 6612 (69.0) | 7570 (67.1) | 9007 (67.7) | 10704 (67.5) | 12704 (67.7) | 15188 (67.0) |
| Methylphenidate | Kuopio | 3614 (73.9) | 4169 (73.7) | 4900 (74.1) | 5892 (74.1) | 7076 (72.1) | 8321 (71.3) |
| Methylphenidate | Oulu | 2283 (73.3) | 2670 (73.9) | 3201 (74.5) | 3775 (73.2) | 4645 (73.1) | 5767 (70.7) |
| Methylphenidate | Tampere | 3559 (78.1) | 4090 (77.0) | 4766 (76.6) | 5584 (75.7) | 6556 (74.1) | 7851 (72.4) |
| Methylphenidate | Turku | 3933 (78.3) | 4303 (75.2) | 4939 (74.2) | 5892 (74.0) | 6696 (72.2) | 7746 (70.0) |
| Lisdexamfetamine | Helsinki | 239 (2.5) | 427 (3.8) | 734 (5.5) | 1104 (7.0) | 1783 (9.5) | 2507 (11.1) |
| Lisdexamfetamine | Kuopio | 119 (2.4) | 258 (4.6) | 377 (5.7) | 603 (7.6) | 897 (9.1) | 1359 (11.7) |
| Lisdexamfetamine | Oulu | 96 (3.1) | 141 (3.9) | 215 (5.0) | 308 (6.0) | 448 (7.0) | 782 (9.6) |
| Lisdexamfetamine | Tampere | 119 (2.6) | 224 (4.2) | 365 (5.9) | 638 (8.6) | 890 (10.1) | 1306 (12.0) |
| Lisdexamfetamine | Turku | 105 (2.1) | 307 (5.4) | 485 (7.3) | 771 (9.7) | 1031 (11.1) | 1411 (12.8) |
| Atomoxetine | Helsinki | 634 (6.6) | 712 (6.3) | 846 (6.4) | 1022 (6.4) | 1120 (6.0) | 1340 (5.9) |
| Atomoxetine | Kuopio | 389 (8.0) | 424 (7.5) | 430 (6.5) | 456 (5.7) | 594 (6.1) | 774 (6.6) |
| Atomoxetine | Oulu | 245 (7.9) | 233 (6.5) | 275 (6.4) | 321 (6.2) | 374 (5.9) | 488 (6.0) |
| Atomoxetine | Tampere | 307 (6.7) | 315 (5.9) | 415 (6.7) | 446 (6.0) | 489 (5.5) | 640 (5.9) |
| Atomoxetine | Turku | 430 (8.6) | 394 (6.9) | 368 (5.5) | 426 (5.3) | 410 (4.4) | 553 (5.0) |
| Dexamfetamine | Helsinki | 0* (0.0) | 159 (1.4) | 258 (1.9) | 293 (1.8) | 381 (2.0) | 454 (2.0) |
| Dexamfetamine | Kuopio | 0* (0.0) | 37 (0.7) | 69 (1.0) | 80 (1.0) | 90 (0.9) | 121 (1.0) |
| Dexamfetamine | Oulu | 0* (0.0) | 23 (0.6) | 39 (0.9) | 55 (1.1) | 61 (1.0) | 78 (1.0) |
| Dexamfetamine | Tampere | 0* (0.0) | 39 (0.7) | 54 (0.9) | 69 (0.9) | 104 (1.2) | 164 (1.5) |
| Dexamfetamine | Turku | 0* (0.0) | 76 (1.3) | 112 (1.7) | 163 (2.0) | 199 (2.1) | 253 (2.3) |
| Guanfacine | Helsinki | 0 (0.0) | 20 (0.2) | 68 (0.5) | 104 (0.7) | 184 (1.0) | 226 (1.0) |
| Guanfacine | Kuopio | 0 (0.0) | 11** (0.2) | 33 (0.5) | 50 (0.6) | 59 (0.6) | 123 (1.1) |
| Guanfacine | Oulu | 0 (0.0) | <5** (0.0) | 9 (0.2) | 19 (0.4) | 21 (0.3) | 40 (0.5) |
| Guanfacine | Tampere | 0 (0.0) | 16 (0.3) | 58 (0.9) | 67 (0.9) | 124 (1.4) | 144 (1.3) |
| Guanfacine | Turku | 0 (0.0) | 16 (0.3) | 50 (0.8) | 58 (0.7) | 71 (0.8) | 103 (0.9) |
| Any ADHD medication | Helsinki | 7081 (73.9) | 8298 (73.5) | 9992 (75.1) | 12014 (75.8) | 14508 (77.3) | 17633 (77.8) |
| Any ADHD medication | Kuopio | 3901 (79.8) | 4547 (80.4) | 5411 (81.9) | 6550 (82.4) | 7977 (81.3) | 9608 (82.4) |
| Any ADHD medication | Oulu | 2483 (79.7) | 2917 (80.8) | 3502 (81.6) | 4210 (81.7) | 5174 (81.4) | 6524 (80.0) |
| Any ADHD medication | Tampere | 3792 (83.2) | 4393 (82.7) | 5221 (83.9) | 6195 (83.9) | 7456 (84.3) | 9114 (84.1) |
| Any ADHD medication | Turku | 4186 (83.4) | 4721 (82.5) | 5508 (82.7) | 6619 (83.1) | 7671 (82.7) | 9003 (81.4) |

* For 2015, not all values marked with an asterisk are true zeroes. Data for these cells are not shown so that no information can be revealed of the total <5 value.

** For 2016, the value <5 has been added to the value of a neighbouring areas so that it cannot be calculated using information presented in other tables.
